# Correlation Between Gestational Weight Gain and Low Back Pain in the Third Trimester: A Cross‐Sectional Study

**DOI:** 10.1155/jp/2063408

**Published:** 2026-06-22

**Authors:** Francisca Paula de Oliveira, Ana Paula Ribeiro, Saulo Gil, Patrícia Colombo-Souza

**Affiliations:** ^1^ Program in Health Sciences, Santo Amaro University—UNISA—São Paulo, São Paulo, Brazil

**Keywords:** gestational weight gain, low back pain, maternal health, third trimester of pregnancy

## Abstract

**Background and Aims:**

Pregnancy involves physiological changes that may lead to low back pain (LBP), especially in the third trimester. Excessive gestational weight gain (GWG) has been suggested as a contributing factor, but evidence in low‐income populations is scarce. This study is aimed at correlating GWG with LBP in the third trimester among postpartum women in São Paulo, Brazil.

**Methods:**

This cross‐sectional study included 53 postpartum women (18–42 years of age) from a public hospital in São Paulo. Data on sociodemographics, GWG, and LBP (visual analog scale, and body map) were collected. Statistical tests included *t*‐test, Mann–Whitney, ANOVA, chi‐square, and linear regression (*α* = 0.05, two‐sided).

**Results:**

Mean age was 28.2 years; 77.3% reported LBP, 53.7% with severe pain (VAS ≥ 6). Mean GWG was 12 kg. Women with severe pain had higher absolute weight gain (14.7 kg vs. 10.3 kg; *p* = 0.028) but no significant difference in weight progression across trimesters. Linear regression showed no causal relationship between pain intensity and GWG alone (*p* = 0.463). Manual workers tended to have more severe pain (70.6% vs. 29.4%; *p* = 0.067).

**Conclusion:**

LBP was highly prevalent (77.3%) and clinically relevant. Higher absolute GWG was associated with greater pain severity, but no independent causal relationship was found. Multifactorial factors, including occupational demands, may mediate this association. Nutritional monitoring and multidisciplinary prenatal care are recommended.

## 1. Introduction

Pregnancy is a period marked by profound physiological changes, including hormonal, musculoskeletal, and metabolic alterations, which are essential for fetal development and maternal adaptation [1]. Among these changes, gestational weight gain stands out as a critical biological phenomenon, influenced by factors such as pre‐pregnancy nutritional status, eating habits, and lifestyle [2]. According to the guidelines of the Institute of Medicine (IOM) (2009), weight gain should be monitored in relation to the pregestational body mass index (BMI) to prevent complications such as gestational diabetes, pre‐eclampsia, and musculoskeletal disorders [3]. However, excess weight has been associated with higher rates of low back pain (LBP), especially in the third trimester, when postural changes—such as increased lumbar lordosis, center of gravity displacement, and ligament laxity—intensify the overload on the spine [4, 5].

Gestational LBP, defined as pain in the lower back region, affects up to 80% of pregnant women, limiting daily activities and impacting quality of life [6, 7]. Its multifactorial etiology includes biomechanical factors (increased uterine pressure and postural adaptations), hormonal factors (relaxin), and psychosocial factors. Studies suggest that weight gain exceeding the IOM recommendations may exacerbate these changes, increasing pressure on the lumbar structures and thereby increasing the risk of disc degeneration [8, 9]. However, evidence on the direct correlation between weight gain and LBP in the third trimester is still scarce and inconsistent, especially in low‐income populations with high social vulnerability. Given this context, this cross‐sectional study sought to investigate the relationship between gestational weight gain and LBP in the third trimester among postpartum women treated at a public hospital in São Paulo. We hypothesized that higher gestational weight gain would be associated with greater prevalence and intensity of LBP, even after considering sociodemographic and occupational factors.

## 2. Methods

### 2.1. Design of the Study and Site

An analytical cross‐sectional study was conducted with postpartum women attending basic health units (UBS) and specialty outpatient clinics (AE) in the southern area of São Paulo, who delivered at the General Hospital of Grajaú (HGG) between August and October 2023.

### 2.2. Population and Eligibility Criteria

Postpartum women from 18 to 42 years old, with gestational age 37 weeks, who had performed at least five prenatal consultations in the UBS and/or AE were included, pregnancy of a single fetus, habitual risk, and agreed to participate in the study by signing the informed consent form (TCLE).

Exclusion criteria include pregnant and puerperal women with relevant orthopedic changes (scoliosis and shortening of the lower limbs). This neurological history causes cognitive or motor impairment of the lower limbs, multiple pregnancy, or refusal to participate in the study.

Sample size was not calculated a priori. A convenience sample of all eligible postpartum women who delivered at the General Hospital of Grajaú during the study period (August to October 2023) and met the inclusion criteria was recruited. This resulted in 53 participants, which reflects the feasibility of data collection in this public hospital setting.

### 2.3. Variables and Collection Instruments

The data collection instrument consisted of a structured questionnaire developed by the researchers, covering sociodemographic, obstetric, and lifestyle variables. For pain assessment, two previously validated instruments were used: the visual analog scale (VAS) for pain intensity (0–10 cm), widely validated for use in Brazilian pregnant and postpartum populations [10, 11], and a body map for pain location, adapted from the Nordic Musculoskeletal Questionnaire and validated for Brazilian adults [12]. Although the complete questionnaire was not formally validated for this specific population, all items were pilot‐tested with 10 postpartum women to ensure clarity and comprehension. No changes were required after piloting.

### 2.4. Independent Variables

Gestational weight gain was calculated using the following equation: ([final weight − initial weight]/initial weight) × 100, being the result expressed as a percentage. The weight, expressed in kilograms, was also analyzed at each consultation with the pregnant woman, with results presented as mean, median, and standard deviation. The pre‐ and postgestational BMIs were classified according to the IOM [13] recommendations in 2009.

### 2.5. Dependent Variables

LBP was verified as present or absent (with or without pain), and the type of pain (if present, whether it was itching or burning) was noted. To assess pain intensity, we used the visual analog scale for pain (VAS), in which puerperal women rated their pain on a scale of 0 to 10, with 0 indicating “no pain” and 10 representing the *worst possible pain*. Cases of moderate low back pain (MLBP) were considered for postpartum women who gave grades from 3 to 5, and severe low back pain (SLBP) for postpartum women who gave grades from 6 to 10 [14].

To locate the pain, a body map was used, featuring a colored drawing of the human body and its muscles on a card, so the puerpera could point to the area where she felt more pain (in the lumbar/pelvic area).

### 2.6. Control Variables

As control variables, sociodemographic data were considered, including age (in whole years), income (minimum wage), occupation (manual/administrative, according to the Brazilian Classification of Occupations, Ministry of Labor), and marital status (with or without a partner).

Still in relation to the control variables, obstetric ones were considered, such as the number of pregnancies/births/abortions, type of delivery (cesarean or vaginal), newborn weight (classified according to WHO) [15], and gestational age (calculated in weeks).

### 2.7. Procedures and Data Sources

Interviews were conducted at the General Hospital of Grajaú, South Zone, City of São Paulo, by a research nurse trained in a private environment during the immediate puerperium. We also used data from the pregnant woman′s card, which contained all information collected during prenatal consultations at the region′s basic health units. It is worth noting that in the event of any reported discomfort, the interview was discontinued.

### 2.8. Statistical Analysis

Statistical analyses were performed using IBM SPSS Statistics Version 27.0 (IBM Corp., Armonk, New York, United States) [16], following the SAMPL guidelines for reporting statistics. Continuous variables were tested for normality using the Shapiro–Wilk test. Normally distributed data were expressed as mean (SD) and compared using Student′s *t*‐test (two‐tailed). Nonnormally distributed data were expressed as median (IQR) and compared using the Mann–Whitney *U* test (two groups) or Friedman′s analysis of variance (repeated measures). Categorical variables were compared using the chi‐square test or Fisher′s exact test, as appropriate. Linear regression analysis was used to examine the relationship between pain intensity (moderate vs. severe) and gestational weight gain, adjusting for pregestational BMI. All tests were two‐sided, with an a priori significance level of *α* = 0.05. Analyses were primarily prespecified; subgroup analyses by pain intensity and occupation were exploratory [17].

Due to the limited sample size (*n* = 53), multivariate regression models adjusting for potential confounders (e.g., occupation, parity, and maternal age) were not feasible, as this would exceed the recommended events per variable (EPV) ratio for logistic regression. Therefore, only bivariate analyses and unadjusted linear regression were performed.

### 2.9. Ethical Aspects

This study was approved by the Ethics Committees of the University of Santo Amaro (UNISA) (Approval No. 4.822.030) and the General Hospital of Grajaú (HGG) (Approval No. 6.155.427) on June 30, 2023. All participants provided written informed consent before enrollment. Data were anonymized and stored on a secure platform.

This cross‐sectional study was reported following the Strengthening the Reporting of Observational Studies in Epidemiology (STROBE) guidelines.

## 3. Results

Fifty‐three puerperal women participated in the study, with a mean age of 28.2 years (18–42 years). The majority (75.5%) had a family income between 1 and 3 minimum wages, 92.4% lived with a partner, and all had a minimum level of education of incomplete secondary education. As for occupation, 70% performed administrative activities (such as secretarial and public service work), and 30% performed manual services, including domestic work.

The majority of participants (98.1%) did not use drugs for continuous use, and 96.2% declared themselves not to be smokers. Of the total, 45.3% were primiparous, whereas 54.7% were multiparous. Most (77.3%) did not report a history of abortion, and 56.6% had vaginal delivery. As for birth weight, 83% of newborns were classified as having adequate weight (2500–3999 g), 15.1% as having low weight (< 2500 g), and 1.9% as weighing 4000 g. The average weight gain during prenatal visits was 12 kg, with a significant increase noted between the 5th and 7th visits in the third trimester (*p* < 0.0001). The analysis by trimester revealed a consistent progression: an average initial weight of 68.2 kg (16.1) at the first consultation (12 weeks) to 80.1 kg (14.3) at the seventh consultation (39 weeks).

All reported pain refers to LBP experienced during the third trimester of pregnancy, assessed retrospectively during the immediate postpartum period. LBP was reported by 77.3% of postpartum women, with 53.7% classified as intense (VAS 6) and 46.3% as moderate (VAS 3–5). All participants with pain described it as “sting,” without reports of “burning.” Workers reported a higher proportion of intense pain (70.6%) than administrative workers (29.4%), although the difference was not statistically significant (*p* = 0.067).

According to the IOM (2009) classification, 56.6% of postpartum women experienced weight gain within the recommended range, whereas 28.3% exceeded the recommended range and 15.1% were below the expected range (Table 1). The individual analysis by percentage of weight gain (relative to pre‐pregnancy weight) showed an average of 19.3% (13.3), ranging from 1% to 66%, with a median of 17.2%. Postpartum women with low initial weight showed significantly higher percentage gain (40.4%, 17.9) compared with those with normal weight (23.3%, 11.7), overweight (12.8%, 4.2), and obesity (10.1%, 5.9) (*p* < 0.01). Significant differences were also observed between normal weight and overweight (*p* < 0.05) and between overweight and obesity (*p* < 0.01), but not between overweight and obesity (*p* = 0.97) (Table 2).

**Table 1:** Evolution of the classification from pregestational to postgestational BMI according to the criteria of the Institute of Medicine (IOM—2009).

BMI Pregestational	Postgestational BMI				Total
	Low weight	Normal weight	Overweight	Obesity	
Low weight	**0**	3	1	1	5 (9.5%)
Normal weight	0	**5**	12	5	22 (41.5%)
Overweight	0	0	**2**	10	12 (22.6%)
Obesity	0	0	0	**14**	14 (26.4%)
Total	0 (0.0%)	8 (15.1%)	15 (28.3%)	30 (56.6%)	53 (100.0%)

*Note:* Data are presented as *n* (vertical %), where the percentage refers to the proportion of women in each pregestational BMI category. For example, 60% (3/5) of women who started pregnancy with low weight remained eutrophic in the postgestational period. The chi‐square statistical test was applied, revealing a significant association between pre‐ and postgestational BMI (*p* value < 0.001*). Bold values along the diagonal represent the number of postpartum women who remained in the same BMI category pre‐ and postgestation (concordance).

Abbreviation: BMI, body mass index.

**Table 2:** Percentage of weight gain in postpartum women. Stratified by pregestational BMI category according to the Institute of Medicine (IOM—2009) criteria.

Pregestational BMI Category	*n*	Weight gain (%)
Low weight (LW)	5	16.0%–66.0%
		Median = 40.3
		Mean = 40.4
Normal weight (NW)	22	3.7%–59.6%
		Median = 22.4
		Mean = 23.3 ± 11.7
Overweight (OW)	12	5.1–18.3%
		Median = 13.8
		Mean = 12.8 ± 4.2
Obesity (OB)	14	1.0%–21.4%
		Median = 9.4
		Mean = 10.1 ± 5.9
*p* value		< 0.001 ^∗^

*Note:* Data are presented as range, median, and mean ± standard deviation (where available). *p* value refers to the one‐way analysis of variance (ANOVA) comparing mean weight gain across all BMI categories. Post hoc analysis (Tukey′s HSD test): LW > NW (*p* < 0.01). OW (*p* < 0.01). And OB (*p* < 0.01). NW > OW (*p* < 0.05) and OB (*p* < 0.01). No significant difference was found between OW and OB (*p* = 0.97).

*p < 0.0001 (ANOVA) for overall comparison among pregestational BMI categories.

The percentage weight gain was higher in mothers with LBP (20.8%) compared with those without pain (14.1%), although the difference was not statistically significant (*p* = 0.068). Similarly, there was no significant difference between moderate (29.4%) and severe (20.4%) pain (*p* = 0.226). Regarding the weight of the newborn, puerperal women with babies of adequate weight (2500–3999 g) had a higher percentage gain (20.1%) compared with those with low weight (14.9%), but the difference was not significant (*p* = 0.291).

Absolute weight gain was higher in postpartum women with low initial weight (19.6 kg 9.0) compared with those with overweight (9.5 kg, 3.2) and obesity (8.7 kg, 4.7) (*p* < 0.01). There was no significant difference between normal weight (13.5 kg, 6.0) and overweight or obesity (*p* = 0.51) (Table 3).

**Table 3:** Absolute weight gain (kg) among postpartum women. Stratified by pregestational BMI category according to the Institute of Medicine (IOM—2009) criteria.

Pregestational BMI category	*n*	Weight gain (kg)
Low weight (LW)	5	8.0–33.0
		Median = 18.0
		Mean = 19.6 ± 9.0
Normal weight (NW)	22	2.0–31.0
		Median = 13.0
		Mean = 13.5 ± 6.0
Overweight (OW)	12	4.0–13.0
		Median = 11.0
		Mean = 9.5 ± 3.2
Obesity (OB)	14	1.0–18.0
		Median = 8.0
		Mean = 8.7 ± 4.7
*p* value		< 0.001 ^∗^

*Note:* Data are presented as range, median, and mean ± standard deviation. *p* value refers to the one‐way analysis of variance (ANOVA) comparing mean weight gain across all BMI categories. Post hoc analysis: Significant differences were found in the following pairwise comparisons: LW > OW (*p* < 0.01) and LW > OB (*p* < 0.01). No significant differences were found between LW and NW, NW and OW, NW and OB, or OW and OB.

*p < 0.01 (ANOVA) for overall comparison among pregestational BMI categories.

The results of Table 4 indicate that total weight gain was significantly higher in the group with severe LBP (14.7 kg, 5.6) than in the group with moderate pain (10.3 kg, 6.8) (*p* = 0.028). When body weight per trimester was analyzed, it did not reveal statistically significant differences between the groups with moderate and severe pain (1st trimester: *p* = 0.879; 2nd trimester: *p* = 0.860; 3rd trimester: *p* = 0.568). This finding suggests that although total weight gain was higher in the group with severe pain (Table 4), the weight progression over the trimesters was similar between the groups. Linear regression analysis did not identify a causal relationship between pain intensity and weight gain (*R* = 0.22; *p* = 0.463 for severe pain) (Table 5).

**Table 4:** Comparison of total body weight gain during pregnancy among postpartum women with moderate (MLBP) to severe (SLBP) low back pain in the third trimester.

Group	*n*	Total body weight gain (kg)	*p* value
Postpartum women with MLBP	29	10.3 ± 6.8	**0.028** ^∗^
Postpartum women with SLBP	12	14.7 ± 5.6	

*Note:* Data are presented as mean ± standard deviation. *p* value refers to the independent student′s *t*‐test. A *p* value < 0.05 was considered statistically significant.

*Statistically significant difference between MLBP and SLBP groups (*p* <0.05, Mann–Whitney *U* test, two‐tailed).

**Table 5:** Simple linear regression analysis between pain intensity and body weight gain among the postpartum groups with moderate to severe low back pain during the last trimester of pregnancy.

Body weight (Kg)	Pain intensity	Body weight gain (Kg)	*t*	*R*	*p*
Postpartum women with MLBP	4.5 ± 0.7	10.3 ± 6.8	0.64	0.16	0.527
Postpartum women with SLBP	6.7 ± 0.8	14.7 ± 5.6	0.74	0.22	0.463

*Note:* Data are presented as mean ± standard deviation. *R*: Pearson correlation coefficient for the simple linear regression between pain intensity and body weight gain. *t*: *t*‐value from the test of the regression slope. *p* value: significance of the correlation; *p* < 0.05 was considered statistically significant.

Abbreviation: LBP, low back pain.

## 4. Discussion

It is important to clarify that all LBP reported by postpartum women in this study refers to symptoms experienced during the third trimester of pregnancy, recalled retrospectively in the immediate postpartum period.

The analysis of the data revealed that 75.5% of the postpartum women were from low‐income socioeconomic strata (D/E classes), with monthly earnings of up to three minimum wages ($520.00), indicating a vulnerability scenario that may compromise access to qualified health services and adherence to adequate prenatal practices. This socioeconomic context, associated with financial limitations, may restrict the acquisition of essential resources for gestational care, such as balanced nutrition and nutritional supplements [18].

It is noteworthy that 92.4% of the participants had a partner, indicating the presence of a socioaffective support network during pregnancy and puerperium. Such support can mitigate psychosocial stressors and promote timely medical assistance, corroborating studies that associate family support with better maternal and child outcomes [19].

All postpartum women had a minimum level of schooling corresponding to incomplete secondary education, a relevant factor, as higher educational levels are correlated with greater adherence to prenatal care, self‐care practices, and understanding of health orientations [20]. Regarding the occupational profile, 70% of participants performed administrative or public service functions, whereas 30% performed manual work. This distribution suggests that physically demanding occupations may increase gestational risks, such as miscarriage, premature birth, and hypertensive disorders, due to physical overload and occupational stress [21]. These findings corroborate the study by Lopes et al. [22], which highlights the influence of external factors on gestational health. In this context, the family support and work activities of pregnant women are identified as factors capable of enhancing the level of stress and risk of complications. It was observed that low social and family support for pregnant women is associated with the presence of high levels of stress, as well as the occupations that pregnant women perform daily.

The distribution between primigestas (45.3%) and multigestas (54.7%) reveals significant nuances in obstetric outcomes and the psychosocial profile of postpartum women. Although multiparity is associated with higher rates of anxiety due to concerns about family dynamics, financial impacts, and previous pregnancies, the prior experience of multigests was advantageous in practices such as breastfeeding at the first postpartum time [23]. This paradox suggests that, despite the stress related to multiparity, accumulated experience can facilitate adaptation to the practical demands of the puerperium. On the other hand, firstborn women, although less exposed to these stressful factors, require more professional support for initial guidance, such as nursing techniques [22, 23].

The high prevalence of healthy habits—such as nonuse of drugs (98%) and smoking abstinence (96%)—was associated with favorable gestational outcomes, including low occurrence of previous abortions (77%) and adequacy of birth weight (83%). These results possibly reflect the quality of prenatal care and the adoption of self‐care practices. In addition, the predominance of vaginal deliveries (56%) reinforces the effectiveness of prenatal care in promoting physiological outcomes, in line with studies that highlight the relationship between qualified assistance and reduction of unnecessary interventions [22, 24, 25]. These findings highlight the importance of policies that integrate health education and equitable access to prenatal services, especially for pregnant women in vulnerable contexts.

The average weight gain of 12 kg, with a significant increase in the third trimester (between the 5th and 7th consultations), aligns with Silva et al.′s [26] recommendations for low‐risk pregnant women, indicating partial adherence to nutritional guidelines. However, there was a trend of higher percentage gain among postpartum women with severe LBP (29.4%) compared with those with moderate pain (20.4%), although without statistical significance (*p* = 0.226). This pattern suggests that even within the recommended limits, individual variations in weight gain can exacerbate musculoskeletal discomfort, possibly due to progressive biomechanical overload. In addition, the study by Silva [26] corroborates the relationship between pregestational BMI and total weight gain, highlighting that pre‐pregnancy weight showed a strong correlation with the final increment (*r* = 0.82), reinforcing the need for customized strategies based on the initial nutritional profile.

Although 56.6% of postpartum women maintained weight gain within the recommended range, 60.4% progressed to higher BMI categories in the postgestational period, indicating challenges in nutritional control (Table 1). This imbalance was more pronounced among pregnant women with newborns of adequate weight (20.1% with excessive gain) than among those with low birth weight (14.9%). However, the difference was not statistically significant (*p* = 0.291). These findings reinforce the importance of continuous monitoring and early interventions to mitigate metabolic and obstetric risks, such as gestational diabetes and macrosomia, associated with inadequate weight gain [2, 27].

The higher weight gain observed in Tables 2 and 3, both in terms of percentage of weight gain and absolute weight, occurred in postpartum women with low pregestational weight, compared with other BMI categories, and may represent an adaptive response to compensate for previous nutritional deficits, thereby ensuring fetal development. This pattern corroborates the findings of Gouvêa et al. [8], which identified a significant association between initial maternal BMI and newborn weight, with eutrophic or overweight pregnant women presenting a higher prevalence of infants with adequate weight. However, contradicting this perspective, Sato and Fujimori [28] demonstrated in a cohort of 228 pregnant women that only total weight gain directly influenced birth weight, without any significant correlation with maternal initial or final BMI. This divergence highlights the complexity of the interaction between previous nutritional status, weight gain, and neonatal outcomes, reinforcing the need for individualized approaches in prenatal care, especially for women with low weight, that may require supplementation and strict monitoring to balance metabolic demands without exacerbating obstetric risks [26–28].

The analysis of labor activities revealed a trend of a higher prevalence of intense pain among manual workers (70.6%) compared with administrative workers (29.4%). However, this difference was not statistically significant (*p* = 0.067), possibly due to sampling limitations or homogeneity between the groups. This qualitative pattern suggests that physically demanding occupations, such as manual services (35.8% of the sample), can exacerbate musculoskeletal overload, predisposing to LBP and pelvic pain. In comparison, administrative activities (64.2%) were associated with lower pain reporting (83.3% of pregnant women without pain belonged to this group). The absence of a direct causal association does not invalidate the clinical relevance of the observed trend, which highlights the need for longitudinal studies with greater statistical power to explore interactions between physical effort, ergonomics, and postural adaptations during pregnancy, aiming to inform occupational health policies for vulnerable populations [29].

Although we observed a trend toward greater weight gain among postpartum women with severe LBP (29.4%) compared with those with moderate pain (20.4%), this difference did not reach statistical significance (*p* = 0.226), suggesting that additional variables may modulate this relationship. According to Salari et al. [29], physiological changes resulting from weight gain—such as a shift in the center of gravity and postural adaptations (e.g., lumbar hyperlordosis and pelvic anteversion)—contribute to musculoskeletal overload, even in pregnant women within the recommended weight range, thereby aggravating low back pain. This biomechanical mechanism is reinforced by Table 4, which identifies a significant difference (*p* = 0.028) in weight gain between postpartum women with severe pain (14.7 ± 5.6 kg) and those with moderate pain (10.3 ± 6.8 kg), particularly in the third trimester, a critical period for increased lumbar pressure due to accelerated uterine growth [27, 28].

A weight gain of 12 kg was a significant factor in the intensity of LBP, especially during the third trimester. In Table 4, postpartum women with severe pain presented with significantly higher weight gain (14.7 ± 5.6 kg; *p* = 0.028) compared with those with moderate pain (10.3 ± 6.8 kg), with 59% of them exceeding the recommended 12 kg, compared with only 21% in the mild pain group. Although the percentage difference (29.4% vs. 20.4%) did not reach statistical significance (*p* = 0.226), biomechanical overload due to weight gain—such as postural changes (hyperlordosis) and increased intra‐abdominal pressure—partially explains the association with pain, as highlighted by Matsuda et al. [30]. These findings suggest that, even within apparently safe parameters, gains close to or above 12 kg can exacerbate musculoskeletal discomfort in susceptible pregnant women.

However, Table 5 shows that linear regression did not identify a significant correlation between pain intensity and weight gain in the MLBP (*p* = 0.527) and SLBP (*p* = 0.463) groups, suggesting that other factors, such as occupation, physical activity, and postural adaptations, mediate this relationship.

The high prevalence of LBP (77.3%) reflects its multifactorial nature, integrating biomechanical, labor‐related, and psychosocial components. The characterization of pain as a “sting” and its intensity (53.7% reported DLI) may be linked not only to weight gain but also to muscle overload and reduced functional capacity, aggravated by repetitive activities. These findings reinforce the importance of prenatal care that includes postural assessments, guidance on safe exercises and strategies for stress management, especially among multiparous women (54.7%), who, although more experienced, face additional challenges related to family dynamics and mental health [31].

As limitations of the study, we can highlight the reduced sample size (*n* = 53) and the possible socioeconomic homogeneity (75.5% of participants belonged to classes D/E), which may have compromised statistical power, especially in stratified analyses. Additionally, the small sample size precluded multivariate adjustment for potential confounders such as occupation, parity, and maternal age, which may have influenced the observed associations. Thus, our findings should be interpreted as exploratory. This explains the lack of significance in associations, such as those between manual labor and LBP (*p* = 0.067), and limits the generalizability of the results to more diverse populations. Retrospective and cross‐sectional data collection make it impossible to establish causal relationships. In addition, variables such as pain intensity, life habits, and weight gain were self‐reported, which introduces risks of memory bias and underreporting, especially in pregnant women with lower education.

Despite sampling and methodological limitations, the findings underscore the urgency of preventive strategies, such as nutritional education and muscle‐strengthening, tailored to contexts of socioeconomic vulnerability where access to specialized care is limited. It is recommended to conduct longitudinal studies that integrate objective postural evaluations and analysis of work habits to uncover causal mechanisms and inform public policies aimed at improving the musculoskeletal health of pregnant women in Brazilian public services.

## 5. Conclusion

Women with higher gestational weight gain presented greater severity of LBP compared with those with lower weight gain. However, the linear regression analysis did not demonstrate a statistically significant correlation between pain intensity and gestational weight gain in isolation. This result suggests that indirect factors or multifactorial interactions may mediate the observed association. Biomechanical overload, such as lumbar hyperlordosis and increased intra‐abdominal pressure, as well as occupational aspects, emerged as key elements in the pathophysiology of pain, reinforcing the complexity of the picture.

## Author Contributions

**Francisca Paula de Oliveira:** investigation, validation, methodology, visualization, writing – original draft, writing – review and editing, formal analysis, data curation. **Ana Paula Ribeiro:** visualization, writing, review, investigation, writing – original draft, validation, methodology, data curation. **Saulo Gil:** investigation, writing – original draft, validation, methodology, visualization, writing – review and editing, formal analysis. **Patrícia Colombo-Souza:** conceptualization, writing – review and editing, visualization, methodology, validation, project administration, supervision, resources.

## Funding

No funding was received for this manuscript.

## Disclosure

Patrícia Colombo‐Souza had full access to all data in this study and takes complete responsibility for the integrity of the data and the accuracy of the data analysis. Patrícia Colombo‐Souza (corresponding author) affirms that this manuscript is an honest, accurate, and transparent account of the study being reported; that no important aspects of the study have been omitted; and that no discrepancies from the study as planned exist (the study was not registered). All authors have read, reviewed, and approved the final content and take full responsibility for the manuscript.

## Ethics Statement

The work was approved by the Ethics Committees of UNISA (Opinion 4.822.030) and the General Hospital of Grajaú HGG (Opinion 6.155.427) on June 30, 2023. Informed consent forms were obtained from all participants, and the data were anonymized and stored on a secure platform to ensure confidentiality.

## Conflicts of Interest

The authors declare no conflicts of interest.

## Data Availability

The data underlying the results of this study are not publicly available due to privacy or ethical restrictions (participants did not consent to public sharing). However, anonymized data may be requested from the corresponding author (Patrícia Colombo‐Souza, pcolombo@prof.unisa.br) upon reasonable request and approval from the institutional ethics committee.
